# A Succinct Overview of Virtual Reality Technology Use in Alzheimer’s Disease

**DOI:** 10.3389/fnagi.2015.00080

**Published:** 2015-05-12

**Authors:** Rebeca I. García-Betances, María Teresa Arredondo Waldmeyer, Giuseppe Fico, María Fernanda Cabrera-Umpiérrez

**Affiliations:** ^1^Life Supporting Technologies (LifeSTech), ETSI Telecomunicaciones, Universidad Politécnica de Madrid, Madrid, Spain

**Keywords:** Alzheimer’s disease, mild cognitive impairment, cognitive rehabilitation, virtual reality, virtual environments

## Abstract

We provide a brief review and appraisal of recent and current virtual reality (VR) technology for Alzheimer’s disease (AD) applications. We categorize them according to their intended purpose (e.g., diagnosis, patient cognitive training, caregivers’ education, etc.), focus feature (e.g., spatial impairment, memory deficit, etc.), methodology employed (e.g., tasks, games, etc.), immersion level, and passive or active interaction. Critical assessment indicates that most of them do not yet take full advantage of virtual environments with high levels of immersion and interaction. Many still rely on conventional 2D graphic displays to create non-immersive or semi-immersive VR scenarios. Important improvements are needed to make VR a better and more versatile assessment and training tool for AD. The use of the latest display technologies available, such as emerging head-mounted displays and 3D smart TV technologies, together with realistic multi-sensorial interaction devices, and neuro-physiological feedback capacity, are some of the most beneficial improvements this mini-review suggests. Additionally, it would be desirable that such VR applications for AD be easily and affordably transferable to in-home and nursing home environments.

## Virtual Reality Technology

Recent brain plasticity theories and findings about the nervous system’s ability to reconstruct cellular synapses as a result of interaction with enriched environments, have spurred new research about memory rehabilitation. Consequently, non-invasive non-pharmacological cognitive rehabilitation (CR) interventions have gained increasing attention in recent years (Cotelli et al., 2012; García-Betances et al., 2014).

Since the introduction of the use of computers for psychological testing over a quarter of a century ago (Riva, 1997), several studies have emphasized the use of virtual environments (VEs) and their ecological validity for neuropsychological assessments (Spooner and Pachana, 2006; Campbell et al., 2009; Tarnanas et al., 2013; Parsons, 2015). VEs have been traditionally defined as “interactive, virtual image displays enhanced by special processing and by non-visual display modalities … to convince users that they are immersed in a synthetic space” (Ellis, 1994). Several software technologies have been introduced into dementia care to assist patients and their families by providing memory aids and educational support (García-Betances et al., 2014). Virtual reality (VR), a recent branch of Information and Communications Technology (ICT), has been suggested for use in some areas of neuropsychology (Rizzo et al., 2001; Schultheis et al., 2002; Rizzo and Kim, 2005; Coyle et al., 2014; Lesk et al., 2014; Shah et al., 2015). Treatment of phobias, stress, and anxiety are characteristic examples of current VR applications in psychotherapy (Esteves and Vidal, 2004; Riva, 2005; Gregg and Tarrier, 2007; Hartanto et al., 2014; McCann et al., 2014; Paliokas et al., 2014; Smahaj and Prochazka, 2014; Fornells-Ambrojo et al., 2015). Other helpful medical uses of VR are surgical training, post-stroke intervention, musculoskeletal recovery, pain mitigation, etc. (Haque and Srinivasan, 2006; Gervasi et al., 2010; Snyder et al., 2011; Imam and Jarus, 2014; Lohse et al., 2014; Pompeu et al., 2014; Trost and Parsons, 2014; Tsoupikova et al., 2015).

Emerging VR applications today address the challenge of diagnosis and cognitive training of mild cognitive impairment (MCI) and dementia patients, concentrating on navigation and orientation, face recognition, cognitive functionality, and other instrumental activities of daily living (IADL) (Jekel et al., 2015). VR exposes cognitively impaired patients to computer-generated VEs providing a sensation of “presence” or “being there,” for the patient to interact with in a multisensory fashion through quasi-naturalistic real-life-like stimuli. Using several perception aspects of psychophysics, mainly visual, tactile, and kinesthetic perceptual sensations, VR offers the possibility of performing activities, tasks, and tests in a VE adaptable to various characteristics and needs of individual patients (Riva, 1997; Baus and Bouchard, 2014; García-Betances et al., 2014). A characteristic of VR, very helpful for Alzheimer’s disease (AD) applications, is the high interaction level that is possible to achieve in a safe VE. Depending on the specific type of VE, patients may interact from egocentric or allocentric points of view (Weniger et al., 2011). The role of egocentric and allocentric abilities in AD have been recently reviewed by Serino et al. (2014). The devices and stimuli used determine the level of interaction. A growing number of devices is available today for interaction (e.g., joysticks, gloves, surfaces, etc.), as well as for stimuli presentation in VEs [e.g., screens, 3D head-mounted displays (HMDs), audio headsets, speakers, etc.].

Slater et al. (2009) described the concept of the level of immersion offered by a VR system by referring to the “fidelity” to real-world sensory experience offered by the system’s displays and tracking in all sensory modalities. Some features of VR systems are most significant when characterizing the immersion level (Slater et al., 2009; Ma and Zheng, 2011; Baus and Bouchard, 2014). They may be reduced to four main types: (a) number of stimulated senses, (b) quantity and level of interactions, (c) synthetic stimuli fidelity, and (d) system’s ability to isolate the user from external stimuli.

Based on the above considerations, three basic levels of system immersion may be defined: (1) non-immersive; (2) semi-immersive; and (3) fully-immersive. In a non-immersive system, the patient interacts with the VE using conventional graphic workstations (PC monitor, keyboard, and mouse) (Costello, 1997; Ma and Zheng, 2011). Virtual tasks played as serious videogames even when displayed on 2D screens are considered for the purpose of this review as “non-immersive” VR, irrespective of the perspective used to look at the scene, whether be it an overhead view or a first-person view, commonly referred to as survey and route perspectives (Markováa et al., 2015). Other more dedicated devices, such as joysticks or gamepads may substitute the mouse. A semi-immersive VR system typically consists of more sophisticated graphics, with larger flat surface displays to present the visual VE (Ma and Zheng, 2011). A fully-immersive VE might consist of huge surrounding projection surfaces, or preferably of 3D displays, such as HMDs, that virtually place the patient inside the VE for the highest level of immersion (Costello, 1997; Baus and Bouchard, 2014).

Immersion plays a crucial role on the subjective sense of “presence.” “Presence” refers to the experience of felling “being there,” that is, how well the VE truly represents a real-world situation, instead of being a simple video viewing experience. “Presence” is strongly related to immersion, since increasing the immersion level induces a higher intensity of the subjective sense of “presence” experienced by the patient (Slobounov et al., 2015). The sense of presence intensity experienced by the patients while performing the required tasks, substantially affects the ensuing behavioral responses (Slobounov et al., 2015). A detailed comparative study of the effect of fully-immersive 3D stereoscopic VEs versus less immersive 2D presentations on brain functions and subsequent behavioral outcomes in VR experiments has been recently published by Slobounov et al. (2015). Health and safety implications of VR use must be taken into serious consideration (Nichols and Patel, 2002), especially when intended for AD patients. Cyber-sickness, a visually induced motion sickness (VIMS) reaction (Keshavarz et al., 2015) that could arise during or after immersion in a VE depending on the level of immersability, should be a matter of concern in clinical settings (Bohil et al., 2011). The possible occurrence and intensity of virtual reality induced sickness symptoms and effects (VRISE) appears to be dependent upon the level of immersion. Experiments have been conducted to comparatively evaluate VRISE in four VR immersion conditions: HMD, PC display, projection screen, and reality theater. In general, higher prevalence of VRISE was found the more immersive the environment is, although a high subject variability was also found (Sharples et al., 2008).

This mini-review does not pretend to be an exhaustive account of all existing applications of VR for AD, rather, it only aims to illustrate, through representative state of the art examples of the most significant types of VR applications, the advantages of using VR for developing a new class of tools in support of the diagnostic assessment of and cognitive training in AD.

## Literature Review Methodology

The overall followed methodology may be divided into two phases: (1) literature search and selection of relevant work and (2) categorization of the selected works. The first phase consisted of an initial computer-based on-line non-systematic literature search, conducted in several high-profile databases, such as: PubMed, Web of Knowledge, IEEExplore, ScienceDirect, and Google Scholar. Only peer-reviewed journal articles in English were considered. Because of conciseness, and considering the relative novelty of the field, the search was limited to years from 2000 to the present. However, a few pre-2000 specific articles were included by reason of their outstanding relevance. VR technology studies and applications related to AD assessment and cognitive intervention were searched for using the following search terms, and combinations thereof: VR, VEs, virtual game, AD, cognitive impairment, CR, and cognitive training.

References cited by the initially retrieved articles became a secondary source for manual selection, and included whenever they contributed significant new information. In addition to the most relevant recent studies of VR applications that involve AD patients, some others aimed at healthy elderly people were included because of their comparative value. Articles dealing with MCI patients were also included since such impairment is often a transition from healthy aging to AD. We excluded those studies and applications in which the devices used to interact with the VE were not clearly described. A few articles whose full-text was not easily accessible were also excluded.

**Figure 1:**
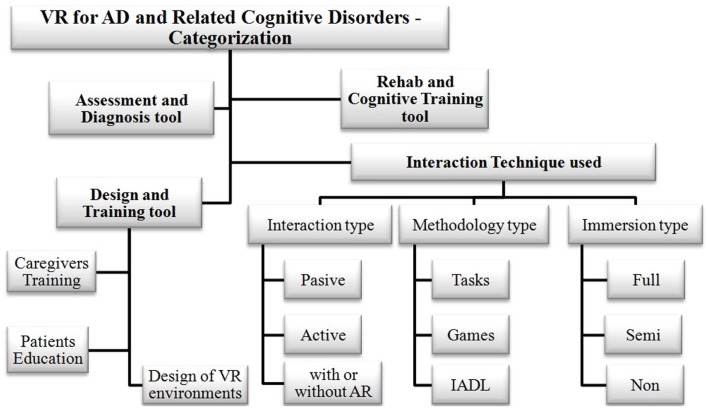
**Categorization of VR technology applications for AD**.

The second methodological phase consisted of categorizing, for later systematic study, the retrieved VR studies and application works, according to the predefined classification schematically portrayed in Figure 1.

## Categorization of VR Applications Used in AD

The potential usefulness and exceptional opportunities of VR systems as valuable ICT tools to assess and train patients in the early stages of AD has been already ascertained by several studies (Schultheis et al., 2002; Gregg and Tarrier, 2007; Déjos et al., 2011; Cotelli et al., 2012; Man et al., 2012; Yamaguchi et al., 2012). For analytical purposes, it seems convenient to classify VR systems according to some functional criteria, as follows: (1) intended purpose (e.g., assessment and diagnosis, cognitive training, patient education, caregivers’ training, etc.); (2) impairment feature it is focused on (e.g., spatial impairment, memory deficit, etc.); (3) methodology employed (e.g., tasks, games, or activities); (4) kind of VE (e.g., desktop, goggles-and-gloves, large screen, virtual room, etc.); and (5) type of interaction technique (e.g., full-immersive, semi-immersive, non-immersive, and passive or active interaction, etc.). Figure 1 presents the schematic representation of such classification.

The pursuit of future advances beyond current VR applications for AD state of the art will benefit from a broad awareness of recent and ongoing VR developments. Such understanding essentially consists of scrutinizing and comparing design and operation specificities that aim to fulfill the intended purpose of particular AD applications, such as the techniques of patient interaction. A useful starting point, particularly regarding design methodology type and immersion level, is Table 1, which lists representative current VR applications for AD and briefly classifies them according to their intended purpose and interaction technique.

**Table 1:** **Recent and current VR applications for AD, classified according to the kind of intended purpose and the type of methodological technique used for interaction**.

VR technology application and/or reference	Participants/users	Focus feature	Intended purpose	Interaction technique used					
				Methodology type			Immersion type		
				Tasks	Games	IADL	Full	Semi	Non
Kalová et al. (2005)	11 early-AD; 27 subjective problems with memory and concentration; 10 healthy controls	Sequential ordering of places, allothetic orientation, spatial navigation, non-verbal episodic memory	**Assessment and diagnosis**	X				X	X
Burgess et al. (2006)	1 early-AD with topographical disorientation; 4 healthy controls	Allocentric spatial memory. Topographical disorientation		X					X
Hort et al. (2007)	21 probable AD; 11 amnestic MCI single domain; 18 amnestic MCI multiple domain; 7 non-amnestic MCI; 8 subjective memory complaints; 26 healthy controls	Spatial memory. Spatial navigation: allocentric and egocentric navigation		X				X	
Lange et al. (2007)	30 mild dementia Alzheimer’s type; 30 healthy controls	Visuospatial and wayfinding orientation		X					X
Cushman et al. (2008)	12 MCI; 14 early-AD; 35 young normal controls; 26 older normal controls	Navigational performance		X					X
Van Schaik et al. (2008)	30 mild to moderate dementia	Evaluation of outdoor environments				X		X	
Zakzanis et al. (2009)	8 healthy young adults; 7 older adults with psychiatric or neurological disorders (2 with probable AD)	Spatial navigation. Spatial memory		X			X		
Laczó et al. (2009, 2010a; 2010b, 2011, 2012)	Amnestic and non-amnestic MCI	Spatial navigation. Hippocampal and non-hippocampal memory impairment		X				X	
Optale et al. (2010)	36 elderly with presence of memory deficits (Verbal Story Recall Test)	Improve memory functions		X			X		
Weniger et al. (2011)	29 amnestic MCI; 29 healthy controls	Egocentric and allocentric memory		X					X
Bellassen et al. (2012)	16 mild AD; 11 frontotemporal lobar degeneration; 24 normal aging	Spatiotemporal navigation. Temporal order memory		X					X
Nedelska et al. (2012)	23 amnestic MCI; 19 mild and moderate AD; 14 healthy controls	Allocentric spatial navigation		X				X	
VREAD, Shamsuddin et al. (2012)	31 healthy elderly and with MCI	Diagnosis of MCI. Cognitive performance. Topographical disorientation		X	X				X
Yeh et al. (2012)	60 senile dementia; 30 healthy controls	Executive functions and memory		X		X	X	X	
Widmann et al. (2012)	15 with AD; 31 healthy controls	Spatial and verbal memory		X				X	
Plancher et al. (2012)	15 amnesic MCI; 15 early to moderate AD; 21 healthy older adults	Episodic memory		X					X
VR-DOT, Tarnanas et al. (2013)	2013: 65 amnestic MCI; 68 mild AD; 72 healthy controls. 2014: 134 with MCI; 75 healthy controls	Executive function. Prospective memory				X		X	
VRAM, Lee et al. (2014)	20 amnestic MCI; 20 mild AD; 20 normal controls	Spatial working memory		X					X
Allain et al. (2014)	24 with AD; 31 healthy elderly controls	IADL functioning		X		X			X
Jebara et al. (2014)	64 young adults; 64 elderly adults	Episodic memory		X				X	
Hofmann et al. (2003)	9 with AD; 9 with major depressive episode; 10 healthy controls	Psychomotor slowing, strategic and critical thinking, cognitive flexibility, problem solving, spatial orientation, delayed recall, long-term memory	**Cognitive training/therapy**	X		X			X
Cognimat, Buss (2009)	6–8 early-AD; 4 healthy controls	Train spatial orientation and working memory		X		X			X
PREVIRNEC, Tost et al. (2009)	Patients with neuropsychological disorders	ADL training		X					X
eGaming, Bartolome et al. (2012)	Patients with neurodegenerative disorders	Cognitive and memory functions			X				X
NeuroRacer, Anguera et al. (2013)	Healthy young and older adults	Enhances cognitive control		X					X
BrightArm, Burdea et al. (2013)	3 with dementia	Cognitive rehabilitation of advanced dementia			X				X
IVIRAGE, Chapoulie et al. (2014)	13 healthy elderly adults	Reminiscence therapy		X				X	
O’Connor et al. (2014)	7 dementia caregivers	On-line support group	**Caregivers’ training**		X				X

### Intended purpose

We have identified three main types of planned goals among the reviewed recent and current VR systems. They may be described as: (1) assessment and diagnosis; (2) cognitive training or therapy; and (3) caregivers’ training. An additional purpose was proposed by Riva et al. (2009) regarding the use of VR systems for designing new tools. They developed “NeuroVR,” an open-source VR platform for assessment and treatment in clinical psychology and neuroscience which allows non-expert users, such as therapists and researchers, to adapt pre-existing VEs to specific clinical or experimental settings needs (Riva et al., 2011). Intended purposes of the recent or current VR applications for AD reviewed here are indicated in Table 1.

### Focal aspects

Recent research has focused on certain specific aspects of AD cognitive impairment features that are generally deemed to be most relevant for VR diagnostic and training purposes. For the sake of the present mini-review, they may be roughly summarized as follows: (1) attention (Kalová et al., 2005; Anguera et al., 2013); (2) executive functions (Yeh et al., 2012; Tarnanas et al., 2013); (3) memory, comprising: non-verbal episodic memory, allocentric and egocentric spatial memory, temporal order memory, prospective memory, short-term, and working memory, etc. (Kalová et al., 2005; Burgess et al., 2006; Optale et al., 2010; Weniger et al., 2011; Bellassen et al., 2012; Shamsuddin et al., 2012; Yeh et al., 2012; Burdea et al., 2013; Tarnanas et al., 2013; Jebara et al., 2014; Lee et al., 2014; Serino and Riva, 2015); (4) orientation, specifically: allothetic, visuospatial, wayfinding, spatial navigation, topographical disorientation, etc. (Kalová et al., 2005; Burgess et al., 2006; Hort et al., 2007; Lange et al., 2007; Cushman et al., 2008; Zakzanis et al., 2009; Nedelska et al., 2012); and also (5) executive functions and IADL (Hofmann et al., 2003; Van Schaik et al., 2008; Buss, 2009; Yeh et al., 2012; Tarnanas et al., 2013, 2014; Allain et al., 2014; Jekel et al., 2015). The justification of the importance of these specific cognitive aspects of AD may be found in the cited references.

Some approaches look at more than one of the above mentioned aspects for better assessment. Examples of this combined focus are: Kalová et al. (2005), Burgess et al. (2006), Hort et al. (2007), Cushman et al. (2008), Laczó et al. (2009), Zakzanis et al. (2009), Weniger et al. (2011), Bellassen et al. (2012), Nedelska et al. (2012), Plancher et al. (2012), and Shamsuddin et al. (2012), likewise, a combination of more than one aspect may be used for more effective training tools (Buss, 2009; Anguera et al., 2013). Frequently, memory and attention are combined with navigation, because navigational impairment is a common manifestation of AD that implies disorders of spatial cognition, spatial memory, and orientation. For example, Bellassen et al. (2012) assessed temporal order memory, as acquired through active navigation (spatiotemporal navigation), to design a sensitive behavioral non-verbal marker of mild AD. Lee et al. (2014) evaluated spatial memory in amnestic MCI (aMCI) patients from results of a virtual route learning environment-labeled virtual radial arm maze (VRAM).

### Interaction techniques

User interaction with VEs and scenarios might involve several methodological modalities. It could consist of playing serious games or performing different tasks or activities (e.g., IADL). Here, we refer to “tasks” and “activities” in reference to VR systems for AD, the term “task” specifically means a particular action that is intended, designed, and established to improve a specific cognitive function, while “activities” involve performing high-level sustained cognitive actions and processes such as: eating, bathing, dressing, shopping, etc. Furthermore, the term “game” refers to activities that are defined by rules and any type of user engagement. Most current VR systems for assessment and diagnosis of AD are based on performing tasks, such as navigation or memorization. Moreover, current VR systems for cognitive training concentrate on performing activities that are related to IADL, such as: cooking, driving, shopping, etc. It has been recently demonstrated that the use of a familiar image-based VE can stimulate recollections of autobiographical memory in healthy elderly subjects (Benoit et al., 2015). The fact that episodic autobiographical memory is impaired in early stages of AD (Seidl et al., 2011), suggests that using such type of VR systems may be helpful for reminiscence rehabilitation of AD patients. Table 1 classifies the reviewed VR systems in terms of the methodology utilized for user interaction.

Recent progress in augmented reality (AR) indicates that this technology will probably become another useful ICT tool for AD. This form of mixed reality enhances a non-synthetic real environment by superimposing some synthetic elements into the users’ perception of that reality (Baus and Bouchard, 2014). In contrast to VR system users, who are exposed to VEs (immersive or not), users of AR applications face real physical locations, upon which AR systems introduce additional virtual elements. Some AR applications have been developed in recent years for medically related purposes, such as phobia therapy (Botella et al., 2005; Wrzesien et al., 2014), and for assessment and treatment of psychological disorders (Giglioli et al., 2015). A few researchers have conducted appraisals of AR applications for cognitive training and rehabilitation of AD patients (Quintana and Favela, 2013). Some examples are the GenVirtual (Correa et al., 2007), BuildAR (Al-Khafaji et al., 2013), and ARCube (Boletsis and Mccallum, 2014).

## Conclusion

We have presented a glimpse at VR applications for diagnostic assessment and cognitive training in MCI and AD. Instead of presenting an exhaustive account of all VR applications currently available, this mini-review has focused on representative state of the art examples of the most significant types, which we have classified into specific categories to aid in their systematic scrutiny. This analysis reveals that most VR applications for AD do not offer today VEs with sufficient levels of immersion or interaction, but simpler non-immersive or semi-immersive VR scenarios.

The present general tendency to develop personalized ICT-based healthcare applications (PMC – Personalized Medicine Coalition, 2014), together with the considerable recent advances in sensor, VR, and 3D technologies, highlights the urgency of continuing the improvement of existing early stage medical VR applications. The improvements should translate in better VR-based applications for AD, with more immersive VEs, based on the latest innovative technologies available (e.g., novel HMDs, 3D smart televisions, etc.). The incorporation of emerging display and interactive technologies will enable innovative designs and implementations of more effective and versatile supportive VR applications for diagnosis and cognitive training of AD patients.

We suggest that future developments of VR cognitive assessment and training applications for MCI and AD should prioritize the specificity of the particular needs of AD patients and their symptoms’ evolution. VR platform designs must be able to incorporate emerging know-how and techniques, not only to better fulfill the intended specific purposes of VR applications for AD, but also to equip those future applications with adequate capacity to supply assistive support to clinicians and caregivers, to significantly contribute to the improvement of the quality of life of MCI and AD patients and their families. Advances in this field should also contemplate providing easy transfer of the applications in a simple and affordable way into in-home and nursing home environments.

Furthermore, a vital feature that could make an important impact on future VR applications for AD usefulness is the capacity to timely gather and transmit relevant information (García-Betances et al., 2014). Such information should consist, not only of ongoing patient performance data, but also of pertinent psycho-physiological data, such as heart rate variability, respiration, ECG, EEG, etc., and any other multimodal information useful for affective (emotional) state recognition (García-Betances et al., 2015) and cognitive stress detection (McDuff et al., 2014). This data gathering feature should also include immediately accessible real-time feedback, to enable computer as well as specialist controlled intervention during the course of the session. Collected data would provide valuable up-to-date information about the patient’s performance evolution, adherence to training and rehabilitation routines, as well as about synchronous physiological reactions of the patient. This feedback capacity could prove to be a valuable tool for implementing other future closed-loop applications, and also become a source of reliable data to build systematic and robust knowledge bases that are indispensable for advancing analytical research and future development.

## Conflict of Interest Statement

The authors declare that the research was conducted in the absence of any commercial or financial relationships that could be construed as a potential conflict of interest.
