# Corrigendum: A succinct overview of virtual reality technology use in Alzheimer’s disease

**DOI:** 10.3389/fnagi.2015.00235

**Published:** 2015-12-14

**Authors:** Rebeca I. García-Betances, María Teresa Arredondo Waldmeyer, Giuseppe Fico, María Fernanda Cabrera-Umpiérrez

**Affiliations:** ^1^Life Supporting Technologies (LifeSTech), ETSI Telecomunicaciones, Universidad Politécnica de Madrid, Madrid, Spain

**Keywords:** Alzheimer’s disease, mild cognitive impairment, cognitive rehabilitation, virtual reality, virtual environments

Due to a misunderstanding, the “last name” of the main author of reference: Giglioli et al., 2015, is a two-word name “Chicchi Giglioli” and not “Giglioli.” Consequently, reference Giglioli et al., 2015 should read:

Chicchi Giglioli, I. A., Pallavicini, F., Pedroli, E., Serino, S., and Giuseppe Riva, G. (2015). Augmented reality: a brand new challenge for the assessment and treatment of psychological disorders, review article. *Comput. Math. Methods Med*. Article ID: 862942.

## Conflict of Interest Statement

The authors declare that the research was conducted in the absence of any commercial or financial relationships that could be construed as a potential conflict of interest.

